# Supplementary material for: Advances in Genetics and Epigenetic Alterations in Alzheimer’s Disease: A Notion for Therapeutic Treatment
Source: Genes (Basel). 2021 Dec 8;12(12):1959. doi: 10.3390/genes12121959 (PMC8700838; doi:10.3390/genes12121959)
Supplement: Supplementary file 1 [file genes-12-01959-s001.zip › genes-1491170-supplementary.pdf]

Advances in genetics and epigenetic alterations in Alzheimer's disease. A notion for therapeutic treatment

SUPPLEMENTARY INFORMATION

Table S1. Potential role and contribution of major causal and genetic disease risk factors for Alzheimer's disease (AD).

Gene	Causal/ risk factor (onset)	Proposed function	Hypothetical contribution to disease
APP	Causal (EOAD)	Cell adhesion, signal transduction, neurite growth and synaptogenesis, signal transduction, calcium metabolism, neuronal protein trafficking along the axon, apoptosis, and the coagulation process.	The physiological function of APP has not yet been fully elucidated and defined, but all studies indicate that it is involved in various processes related to metabolism and neuronal communication, among others [1, 2]. There are more than 25 missense variants of APP, most of which are associated with EOAD. These mutations are located within the Aβ domain or in close proximity to the Aβ sequence, which results from the sequential cleavage of APP by the enzymes BACE1 and γ-secretase. Some of these mutations are related to CAA. Recently, BACE2 was found to be involved in APP cleavage at the β-site within the Aβ-domain and to contribute to the exacerbation of AD pathogenesis.
PSEN1	Causal (EOAD)	PSEN1 could function as a catalytic subunit of the γ-secretase complex and promote cleavage of APP-CTFs	Point mutations in PSEN1 are the cause of EOAD. 326 mutations have been reported in the Alzforum database.
PSEN2	Causal (EOAD)	Regulation of APP processing cleavage and NOTCH receptor protein cleavage.	Point mutations in PSEN2 are the cause of EOAD. Sixty-eight mutations have been reported in the Alzforum database.

APOE	Risk factor<br><br>(EOAD, LOAD)	Lipid metabolism (VLDL)<br><br>β-amyloid metabolism?	There are three isoforms that confer differential susceptibility to AD: ApoE3 is most common in white populations (78%) and is thought to have neutral effects. APOE-ε2 is a rare variant (5% frequency in the population) and is considered a protective factor for AD, which could affect men and women differently. APOE-ε4 occurs in less than 20% of the population but has a prevalence of 50% in individuals with AD, and the risk varies depending on whether the individual carries one or two APOE-ε4 alleles.
CR1	Risk factor<br><br>(LOAD)	Aβ metabolism (clearance)<br><br>Immune system function (antigen-binding protein)	Decreased expression of this gene has been associated with several diseases such as gallbladder carcinoma and sarcoidosis. Some studies have identified genetic polymorphisms associated with neurodegenerative processes likely related to the pathogenesis of AD [3, 4]. R1 has also been shown to mediate susceptibility to AD via clearance of amyloid Aß in the brain [5] and interactions of CR1 variants with APOE-ε4 have been observed to be strongly associated with cognitive impairment [6].
BIN1	Risk factor<br><br>(LOAD)	Endocytic membranes, pathways, and T-tubule conformation.	Recent work has shown that BIN1 can interact with the tau protein in human neuroblastoma cells and mouse brain, contributing to the risk of AD by altering neuronal degeneration [7].
CLU	Risk factor<br><br>(LOAD)	Cholesterol/lipid metabolism (binding and transport)<br><br>Aβ-metabolism (clearance).<br><br>Regulation of the function of the immune response	Identified as the third most important risk locus for the development of LOAD. Although some risk-increasing polymorphisms have been isolated at this locus [8–10], the pathogenic mechanisms of these variants remain unclear.
SORL1	Causal/Risk factor<br><br>(EOAD, LOAD)	Metabolism of APP (trafficking in endocytic pathways and sorting associated with Aβ).	Common and rare variants in SORL1 have been linked to the pathogenesis of EOAD and LOAD and confer low to moderate risk by increasing Aβ secretion and altering the processing of APP [11 – 13].
PICALM	Risk factor<br><br>(EOAD, LOAD)	Aβ-metabolism (deposition).<br><br>Regulation of membrane metabolism (cellular transport, endocytosis-clathrin pathways).	It encodes a clathrin protein that may contribute to the formation of amyloid plaques in the brain [14]. Several polymorphisms are significantly associated with AD and Aβ-peptide transport in the brain [15]. It has been suggested that expression of PICALM in the microvasculature reduces the risk of AD [16].

TREM2	Risk factor<br><br>(EOAD, LOAD)	Regulation of cell-mediated immunity (triggers activation of immune response function in macrophages and dendritic cells; inflammatory response).<br><br>Expressed in microglia	Point mutations in TREM2 are the cause of EOAD[17]. 11 mutations have been reported in the Alzforum database.
ABCA7	Risk factor<br><br>(LOAD)	Cholesterol/lipid metabolism (transmembrane trafficking; phagocytosis promoter by macrophages of apoptotic cells).<br><br>Regulation of APP processing via BACE1 and Aβ metabolism (endocytic pathways).<br>Immune system function (lipid homeostasis in cells of IS).<br><br>Highest expression in microglia	Loss of function of ABCA7 alters amyloid processing. VNTR disrupts ABCA7 splicing and is associated with an increased risk of AD [18]. Rare ABCA7 variants are associated with familial AD [19].
MS4A6A	Risk factor<br><br>(interaction, variable)	Signal Transduction<br><br>Four-span transmembrane protein	The MS4A family functions as a chemosensor for a variety of exogenous and endogenous ligands. SNPs in the MS4A gene region have a dose-dependent effect on LOAD risk [20, 21]. MS4A6A genotypes are associated with AD -specific brain structures [22].
MS4A4E	Risk factor<br><br>(interaction, variable)	Four-span transmembrane protein	AD risk variants in MS4A4E affect soluble TREM2 content in CSB, Together with CLU, MS4A4E modulates risk AD.
CD33	Risk factor	Transmembrane protein/cell interaction.	CD33 is a myeloid cell receptor that is highly expressed in AD brains (microglia and macrophages), where it is involved in modulating microglial activation and regulating Aβ metabolism by inhibiting Aβ clearance. CD33 is also involved in immune cell growth and adhesion processes, as well as in the induction of apoptosis [23].

TOMM40	Risk factor<br><br>(variable)	TOMM40 (Translocase of the Outer Mitochondrial Membrane 40)	The TOMM40 gene is located near APOE and is imbalanced with it. Polymorphisms in TOMM40 intron 6 are associated with AD. Variants in TOMM40 and APOE increase the risk of AD in different populations and ethnicities [24, 25].
CD2AP	Risk factor<br><br>(EOAD, LOAD)	Regulation of actin cytoskeleton dynamics/membrane trafficking metabolism (clearance).<br><br>Highest expression in microglia	CD2AP mRNA levels are decreased in the blood of sporadic AD patients. Common variants in CD2AP have been significantly associated with EOAD.
MAPT	Risk factor<br><br>(EOAD, LOAD)	It promotes microtubule assembly and stability and is thought to be involved in the formation and maintenance of neuronal polarity.	It encodes the tau protein, and mutant variants have been linked to several neurodegenerative diseases, including AD and dementia, which contribute to the risk of LOAD [26, 27]. Mutations in the MAPT gene encoding tau can lead to frontotemporal dementia with chromosome 17-linked parkinsonism (FTDP-17)[28].

Table S2. Results of a quick search of the NCBI PubMed database using the words "epigenome and Alzheimer". 264 results were found of which only original articles were selected (review articles were excluded).

Epigenomic signal	AD model	Main finding	Reference
Histone acetylation	Post-mortem human prefrontal cortices	Tau pathology, but not amyloid-β pathology, affects histone acetylation.<br>In neurons, altered transcription occurs due to tau-induced chromatin remodeling. Tau-related chromatin changes exhibit spatial patterns.	[29]
DNA methylation	Post-mortem human brain tissue (AD Braak stage progression).	Identification of genes with cell type-specific methylation signatures and documentation of age-related differential methylation dynamics in neurons (CLU, NCOR2 and SYNJ2) or glia (CXXC5, RAI1 and INPP5A).<br>Several DNA methylation signals of neuronal (HOXA3) or glial origin (ANK1) associated with AD were validated.	[30]
DNA methylation	AβPPswe/PS-1 mice	Dlg4/PSD95, a protein involved in neuronal plasticity and memory, exhibits increased expression during development regulated by histone marks that have a major impact on several processes of hippocampal plasticity and neurons.	[31]
DNA methylation	Postmortem human brain tissue (middle temporal gyrus) and blood samples in AD patients.	Gene regulatory network (GRN) analysis predicts altered expression of IL6 and SIAH1 genes in brain tissues influenced by methylation and hydroxymethylation.<br>In blood, WNT3A is the leading gene in the reference network.<br>In both tissues, a common DMR is identified near the transcriptional starting point of the gene OXT, which encodes the neuropeptide oxytocin involved in neuromodulation of social behavior.	[32]
Histone acetylation and methylation	Human cortical brain tissue (microglia, neuronal and oligodendrocyte)	A subset of the variants identified by GWAS could act on super-enhancers that would affect gene expression. Knockdown of a targeted microglial enhancer carrying AD risk variants suppresses BIN1 gene expression in microglia but not in neurons or astrocytes.	[33]
DNA methylation	DNA methylation of induced pluripotent stem cells (iPSCs) from AD patients and healthy controls. One normal cell line, one LOAD line (APOE4) and at least two EOAD cell lines (PSEN1, PSEN2) are included.	Different DMRs of 5mC, 5hmC, and 5fC/caC are identified during differentiation of iPSCs into neurons in both normal cells and PSEN2.	[34]
DNA methylation	Whole blood DNA from trisomy 21 (T21) patients, non-dementia patients and AD dementia patients.	In both T21 and AD patients, at least 6 hypermethylated sites occur compared to healthy individuals. One of them is located in the ADAM10 promoter region.	[35]
DNA methylation	Blood DNA from an international population of	It has been observed that genetic factors contribute to differential DNA methylation in	[36]

	eleven cohorts totaling 3337 individuals.	the hippocampus. Methylation at these sites alters the expression of genes required for hippocampal function and metabolic regulation.	
mtDNA methylation	Postmortem PFC samples and Human cell lines HEK293T and A549.	mtDNA methylation is negatively correlated with mitochondrial gene expression and is modulated by methyltransferase (DNMT3A), which is required for the maintenance of methylation in neurons.	[37]
DNA methylation	E4 and E3 mice with high fat diet (HFD)	E4 HFD mice exhibit a unique DNA methylation profile in the hippocampus. They find that HFD-induced deficits in learning and spatial memory, but not object recognition, are more pronounced in E4 mice.	[38]
DNA methylation	DNA methylation in the entorhinal cortex of the brain (EC) Samples from the MRC London Neurodegenerative Diseases Brain Bank.	The ANK1 gene exhibits differential DNA methylation at AD.<br>Abnormal DNA methylation changes in WNT5B are associated with AD neuropathology.	[39]
DNA methylation	Peripheral blood samples from patients with MCI and normal control subjects.	Discovery of DMRs: two within SEPT8 and TMEM232 on chromosome 5, one within SLC17A8 on chromosome 12, and another within ALOX12 on chromosome 17.<br>Functional methylation analysis identifies four groups of genes (modules) that are significantly hypomethylated in affected individuals compared to controls: RIN3, CTSG, SPEG and UBE2L3.	[40]
Histone acetylation and methylation	Female triple transgenic (3xTg-AD) mice	The DNA methylation level at the promoter of the Txnip gene in the brain is significantly lower in 3xTg- AD compared to wild type.<br>The level of DNA methylation at the CTCF region of the Txnip gene promoter is significantly lower in the cerebellum and significantly higher in the spleen of 3xTg- AD mice compared to wild-type controls.	[41]
DNA methylation	Postmortem brains of age-matched normal controls and AD patients.	Methylation levels in the promoter regions of the BRCA1 and AURKC genes are upregulated in AD brains.<br>Dysfunction of BRCA1 results in impaired DNA integrity.	[42]
DNA methylation	Four brain regions: Hippocampus, cerebellum, EC and dorsolateral PFC of donor controls and patients with late stages AD.	Identify 858 robust DMRs in up to 772 putative genes.<br>Identify CpGs near ANK1 and MYO1C genes with AD.<br>The region-dependent and most significant effect is observed for a DNA methylation site near ANK1, which is more methylated in subjects with AD in the dorsolateral PFC, EC and hippocampus, but less methylated in the cerebellum.	[43]
DNA methylation	APP/PS1 mouse	PM20D1 is involved in lipid metabolism and is an important methylation and expression locus	[44]

		located within a AD -risk associated haplotype.	
Histone acetylation	THY-Tau22 mouse	The histone acetyltransferase (HAT)-activating molecule CBP/p300 (CSP -TTK21) is capable of acetylating nuclear chromatin in mouse brain. It shows a specific decrease in acetylation levels in the hippocampus of THY -Tau22 mice, and CSP -TTK21 significantly restores this signature by enhancing long-term spatial memory storage.	[45]
DNA methylation	Postmortem brain tissue from patients with AD; dementia with Lewy bodies (DLB); Huntington's disease (HD); Parkinson's disease vascular dementia and non-demented control subjects.	Significantly increased levels in AD cases compared to controls AT eight ANK1 CpG sites in the ERC and seven ANK1 CpG sites in theSTG. Changes in ANK1 DNA methylation in the cerebellum are reported for the first time. DNA hypermethylation of ANK1 in the ERC is observed only in DLB cases with coexisting AD pathology.	[46]
DNA methylation	Postmortem hippocampal samples from AD patients and control subjects.	DNA methylation levels correlate significantly with exposure to p-tau deposition. Genomic loci that strongly overlap in regulatory regions are significantly hypermethylated in AD compared to healthy patients. DMGs are associated with neuronal development and neurogenesis.	[47]
Histone acetylation and methylation	APP/PS1 mice	Overall, no changes in histone marks over time in APP/PS1 and wild-type mice.<br>Age-related changes in histone marks are observed in wild-type mice.	[48]
DNA methylation	Post-mortem PFC of normal subjects and AD patients (LOAD).	They found 504 differentially methylated positions (DMPs), of which 487 positions had increased methylation levels and 17 positions had reduced methylation levels compared to controls AD.<br>The DMPs are mainly located in the HOXA3, GSTP1, CXXC1-3 and BIN1 genes.	[49]
DNA methylation	Post-mortem brains of AD patients and healthy controls.	DNA methylation (H3K9me3) is upregulated in the temporal cortex of patients with sporadic AD.	[50]
DNA methylation	Peripheral blood monocytes from healthy controls and AD patients. Cortex samples from 4 healthy subjects and 4 AD patients.	Hypomethylation of the TNF-α promoter region in the cerebral cortex of AD patients, while similar levels of methylation are found in control groups and in blood samples from AD patients.	[51]
DNA methylation	Brain tissue from 147 individuals drawn from the Mount Sinai Alzheimer's and Schizophrenia Brain Bank.	DNA hypermethylation in a 48 kb region of the HOXA gene is associated with neuropathology of AD in human cerebral cortex and cortical neuropathology of HOXA3.	[46]
DNA methylation	DNA methylation DNA from postmortem prefrontal cortical corx tissue from patients with AD and controls.	325 genes with differentially hydroxymethylated loci were identified.<br>Gene enrichment analysis in ontology reveals biological processes related to the development of neuronal projections and neurogenesis.	[52]

DNA methylation	Postmortem samples from STG patients with late onset AD and control subjects.	A total of 17,895 CpG sites were preliminarily identified as differentially methylated between AD and control subjects, including 11,822 and 6,073 hyper- and hypomethylated CpGs, respectively. Hypermethylation was mainly detected in genes regulating cell cycle progression.	[53]
DNA methylation	Peripheral blood (PB) samples from cognitively normal (CN), MCI, and AD patients.	DMPs with the strongest association with MCI compared to CN are annotated with CLIP4 and those most strongly associated with AD compared to CN are annotated with FAM8A1.	[54]
DNA methylation	LOAD patients and age- and sex-matched controls.	Significantly higher levels of D-loop methylation are observed in heterozygous MTRR 66AG carriers compared to wild-type MTRR 66AA individuals. Stratification of the population into AD and control subgroups shows that even in the AD subgroup, carriers of the DNMT3A AA genotype have significantly higher levels of D-loop methylation than GG or GA carriers. They suggest that MTRR and DNMT3A polymorphisms affect mitochondrial DNA methylation.	[55]
Blood DNA methylation	Blood samples from individuals before dementia diagnosis and cognitively healthy controls.	The biggest difference in methylation is the lower methylation in diagnosed dementia compared to controls.<br>DMRs are found together when blood samples are analysed before and after diagnosis. Genes affected by these DMRs include GULP1, SORCS3, PIEZO2, DNAH14, RIBC2, FOXG1, HOXC5, EPHA6, HOXA7, SYN3, IRX4, NOS1, LOC101929268, MARCH3, and ADAM12.	[56]

References

[1] Guo, Q.; Wang, Z.; Li, H.; Wiese, M.; Zheng, H. APP Physiological and Pathophysiological Functions: Insights from Animal Models. *Cell Res.*, **2012**, *22* (1), 78–89. https://doi.org/10.1038/cr.2011.116.

[2] Aydin, D.; Weyer, S. W.; Müller, U. C. Functions of the APP Gene Family in the Nervous System: Insights from Mouse Models. *Exp. Brain Res.*, **2012**, *217* (3), 423–434. https://doi.org/10.1007/s00221-011-2861-2.

[3] Brouwers, N.; Van Cauwenberghe, C.; Engelborghs, S.; Lambert, J.-C.; Bettens, K.; Le Bastard, N.; Pasquier, F.; Montoya, A. G.; Peeters, K.; Mattheijssens, M.; et al. Alzheimer Risk Associated with a Copy Number Variation in the Complement Receptor 1 Increasing

C3b/C4b Binding Sites. *Mol. Psychiatry*, **2012**, *17* (2), 223–233. https://doi.org/10.1038/mp.2011.24.
[4] Crehan, H.; Holton, P.; Wray, S.; Pocock, J.; Guerreiro, R.; Hardy, J. Complement Receptor 1 (CR1) and Alzheimer's Disease. *Immunobiology,* **2011**, *217*, 244–250. https://doi.org/10.1016/j.imbio.2011.07.017.
[5] Fonseca, M.; Chu, S.-H.; Pierce, A.; Brubaker, W.; Hauhart, R.; Mastroeni, D.; Clarke, E.; Rogers, J.; Atkinson, J.; Tenner, A. Analysis of the Putative Role of CR1 in Alzheimer's Disease: Genetic Association, Expression and Function. *PLoS One,* **2016**, *11*, e0149792. https://doi.org/10.1371/journal.pone.0149792.
[6] Keenan, B.; Shulman, J.; Chibnik, L.; Raj, T.; Trần, Đ.; Sabuncu, M.; Allen, A.; Corneveaux, J.; Hardy, J.; Huentelman, M.; et al. A Coding Variant in CR1 Interacts with APOE- 4 to Influence Cognitive Decline. *Hum. Mol. Genet.*, **2012**, *21*, 2377–2388. https://doi.org/10.1093/hmg/dds054.
[7] Tan, M.-S.; Yu, J.-T.; Tan, L. Bridging Integrator 1 (BIN1): Form, Function, and Alzheimer's Disease. *Trends Mol. Med.*, **2013**, *19*. https://doi.org/10.1016/j.molmed.2013.06.004.
[8] Szymanski, M.; Wang, R.; Bassett, S.; Avramopoulos, D. Alzheimer's Risk Variants in the Clusterin Gene Are Associated with Alternative Splicing. *Transl. Psychiatry*, **2011**, *1*. https://doi.org/10.1038/tp.2011.17.
[9] Gu, H.; Wei, X.; Chen, S.; Kurz, A.; Müller, U.; Gasser, T.; Dodel, R.; Farlow, M.; Du, Y. Association of Clusterin Gene Polymorphisms with Late-Onset Alzheimer's Disease. *Dement. Geriatr. Cogn. Disord.*, **2011**, *32*, 198–201. https://doi.org/10.1159/000331276.
[10] Shuai, P.; Liu, Y.; Lu, W.; Liu, Q.; Li, T.; Gong, B. Genetic Associations of CLU Rs9331888 Polymorphism with Alzheimer's Disease: A Meta-Analysis. *Neurosci. Lett.*, **2015**, *591*. https://doi.org/10.1016/j.neulet.2015.02.040.
[11] Webster, J.; Myers, A.; Pearson, J.; Craig, D.; Hu-Lince, D.; Coon, K.; Zismann, V.; Beach, T.; Leung, D.; Bryden, L.; et al. Sorl1 as an Alzheimer's Disease Predisposition Gene? *Neurodegener. Dis.*, **2008**, *5*, 60–64. https://doi.org/10.1159/000110789.
[12] Mccarthy, J.; Saith, S.; Linnertz, C.; Burke, J.; Hulette, C.; Welsh-Bohmer, K.; Chiba-Falek, O. The Alzheimer's Associated 5 ' Region of the SORL1 Gene Cis Regulates SORL1 Transcripts Expression. *Neurobiol. Aging*, **2010**, *33*, 1485.e1-8. https://doi.org/10.1016/j.neurobiolaging.2010.10.004.
[13] Vardarajan, B.; Zhang, Y.; Lee, J.; Cheng, R.; Bohm, C.; Ghani, M.; Reitz, C.; Reyes-Dumeyer, D.; Shen, Y.; Rogaeva, E.; et al. Coding Mutations in SORL1 and Alzheimer Disease. *Ann. Neurol.*, **2014**, *77*. https://doi.org/10.1002/ana.24305.
[14] Cuccaro, M.; Carney, R.; Zhang, Y.; Bohm, C.; Kunkle, B.; Vardarajan, B.; Whitehead, P.; Cukier, H.; Mayeux, R.; George-Hyslop, P.; et al. SORL1 Mutations in Early- and Late-Onset Alzheimer Disease. *Neurol. Genet.*, **2016**, *2*, e116. https://doi.org/10.1212/NXG.0000000000000116.
[15] Xiao, Q.; Gil, S.-C.; Yan, P.; Wang, Y.; Han, S.; Gonzales, E.; Perez, R.; Cirrito, J.; Lee, J.-M. Role of Phosphatidylinositol Clathrin Assembly Lymphoid-Myeloid Leukemia (PICALM) in Intracellular Amyloid Precursor Protein (APP) Processing and Amyloid Plaque Pathogenesis. *J. Biol. Chem.*, **2012**, *287*, 21279–21289. https://doi.org/10.1074/jbc.M111.338376.
[16] Xu, W.; Tan, L.; Yu, J.-T. The Role of PICALM in Alzheimer's Disease. *Mol. Neurobiol.*, **2015**, *52* (1), 399–413. https://doi.org/10.1007/s12035-014-8878-3.

[17] Piccio, L.; Deming, Y.; Del-Aguila, J.; Ghezzi, L.; Holtzman, D.; Fagan, A.; Fenoglio, C.; Galimberti, D.; Borroni, B.; Cruchaga, C. Cerebrospinal Fluid Soluble TREM2 Is Higher in Alzheimer Disease and Associated with Mutation Status. *Acta Neuropathol.*, **2016**, *131*. https://doi.org/10.1007/s00401-016-1533-5.
[18] De Roeck, A.; Duchateau, L.; Van Dongen, J.; Cacace, R.; Bjerke, M.; Van den Bossche, T.; Cras, P.; Vandenberghe, R.; De Deyn, P. P.; Engelborghs, S.; et al. An Intronic VNTR Affects Splicing of ABCA7 and Increases Risk of Alzheimer's Disease. *Acta Neuropathol.*, **2018**, *135* (6), 827–837. https://doi.org/10.1007/s00401-018-1841-z.
[19] May, P.; Pichler, S.; Hartl, D.; Bobbili, D. R.; Mayhaus, M.; Spaniol, C.; Kurz, A.; Balling, R.; Schneider, J. G.; Riemenschneider, M. Rare <Em>ABCA7</Em> Variants in 2 German Families with Alzheimer Disease. *Neurol. Genet.*, **2018**, *4* (2), e224. https://doi.org/10.1212/NXG.0000000000000224.
[20] Ma, Y.; Jun, G. R.; Chung, J.; Zhang, X.; Kunkle, B. W.; Naj, A. C.; White, C. C.; Bennett, D. A.; De Jager, P. L.; Consortium, A. D. G.; et al. CpG-Related SNPs in the MS4A Region Have a Dose-Dependent Effect on Risk of Late-Onset Alzheimer Disease. *Aging Cell*, **2019**, *18* (4), e12964–e12964. https://doi.org/10.1111/acel.12964.
[21] Hollingworth, P.; Harold, D.; Sims, R.; Gerrish, A.; Lambert, J.-C.; Carrasquillo, M.; Abraham, R.; Hamshere, M.; Pahwa, J.; Moskvina, V.; et al. Common Variants at ABCA7, MS4A6A/MS4A4E, EPHA1, CD33 and CD2AP Are Associated with Alzheimer's Disease. *Nat. Genet.*, **2011**, *43*, 429–435. https://doi.org/10.1038/ng.803.
[22] Ma, J.; Zhang, W.; Tan, L.; Wang, H.-F.; Wan, Y.; Sun, F.-R.; Tan, C.-C.; Yu, J.-T.; Tan, L.; Initiative, A. D. N. MS4A6A Genotypes Are Associated with the Atrophy Rates of Alzheimer's Disease Related Brain Structures. *Oncotarget*, **2016**, *7* (37), 58779–58788. https://doi.org/10.18632/oncotarget.9563.
[23] Zhao, L. CD33 in Alzheimer's Disease – Biology, Pathogenesis, and Therapeutics: A Mini-Review. *Gerontology*, **2019**, *65* (4), 323–331. https://doi.org/10.1159/000492596.
[24] Zhu, Z.; Yang, Y.; Xiao, Z.; Zhao, Q.; Wu, W.; Liang, X.; Luo, J.; Cao, Y.; Shao, M.; Guo, Q.; et al. TOMM40 and APOE Variants Synergistically Increase the Risk of Alzheimer's Disease in a Chinese Population. *Aging Clin. Exp. Res.*, **2021**, *33* (6), 1667–1675. https://doi.org/10.1007/s40520-020-01661-6.
[25] Deters, K. D.; Mormino, E. C.; Yu, L.; Lutz, M. W.; Bennett, D. A.; Barnes, L. L. TOMM40-APOE Haplotypes Are Associated with Cognitive Decline in Non-Demented Blacks. *Alzheimer's Dement.*, **2021**, *17* (8), 1287–1296. https://doi.org/https://doi.org/10.1002/alz.12295.
[26] Allen, M.; Kachadoorian, M.; Quicksall, Z.; Zou, F.; Chai, H.; Younkin, C.; Crook, J.; Pankratz, V.; Carrasquillo, M.; Krishnan, S.; et al. Association of MAPT Haplotypes with Alzheimer's Disease Risk and MAPT Brain Gene Expression Levels. *Alzheimers. Res. Ther.*, **2014**, *6*. https://doi.org/10.1186/alzrt268.
[27] Desikan, R.; Schork, A.; Wang, Y.; Witoelar, A.; Sharma, M.; Mcevoy, L.; Holland, D.; Brewer, J.; Chen, C.-H.; Thompson, W.; et al. Genetic Overlap between Alzheimer's Disease and Parkinson's Disease at MAPT Locus. *Mol. Psychiatry*, **2015**, *20*. https://doi.org/10.1038/mp.2015.6.
[28] Strang, K. H.; Golde, T. E.; Giasson, B. I. MAPT Mutations, Tauopathy, and Mechanisms of Neurodegeneration. *Lab. Invest.*, **2019**, *99* (7), 912–928. https://doi.org/10.1038/s41374-019-0197-x.
[29] Klein, H.-U.; McCabe, C.; Gjoneska, E.; Sullivan, S. E.; Kaskow, B. J.; Tang, A.;

Smith, R. V; Xu, J.; Pfenning, A. R.; Bernstein, B. E.; et al. Epigenome-Wide Study Uncovers Large-Scale Changes in Histone Acetylation Driven by Tau Pathology in Aging and Alzheimer's Human Brains. *Nat. Neurosci.*, **2019**, *22* (1), 37–46. https://doi.org/10.1038/s41593-018-0291-1.
[30] Gasparoni, G.; Bultmann, S.; Lutsik, P.; Kraus, T.; Sordon, S.; Vlcek, J.; Dietinger, V.; Steinmaurer, M.; Haider, M.; Mulholland, C.; et al. DNA Methylation Analysis on Purified Neurons and Glia Dissects Age and Alzheimer's Disease-Specific Changes in the Human Cortex. *Epigenetics Chromatin*, **2018**, *11*. https://doi.org/10.1186/s13072-018-0211-3.
[31] Bustos, F.; Ampuero, E.; Jury, N.; Aguilar, R.; Falahi, F.; Toledo, J.; Ahumada, J.; Lata, J.; Cubillos, P.; Henriquez, B.; et al. Epigenetic Editing of the Dlg4/PSD95 Gene Improves Cognition in Aged and Alzheimer's Disease Mice. *Brain*, **2017**, *140*. https://doi.org/10.1093/brain/awx272.
[32] Lardenoije, R.; Roubroeks, J.; Pishva, E.; Leber, M.; Wagner, H.; Iatrou, A.; Smith, A.; Smith, R.; Eijssen, L.; Kleineidam, L.; et al. Alzheimer's Disease-Associated (Hydroxy)Methylomic Changes in the Brain and Blood. *Clin. Epigenetics*, **2019**, *11*. https://doi.org/10.1186/s13148-019-0755-5.
[33] Nott, A.; Holtman, I.; Coufal, N.; Schlachetzki, J.; Yu, M.; Hu, R.; Han, C.; Pena, M.; Xiao, J.; Wu, Y.; et al. Brain Cell Type–Specific Enhancer-Promoter Interactome Maps and Disease Risk Association. *Science (80-.).*, **2019**, *366*, eaay0793. https://doi.org/10.1126/science.aay0793.
[34] Fetahu, I.; Ma, D.; Rabidou, K.; Argueta, C.; Smith, M.; Liu, H.; Wu, F.; Shi, Y. Epigenetic Signatures of Methylated DNA Cytosine in Alzheimer's Disease. *Sci. Adv.*, **2019**, *5*, eaaw2880. https://doi.org/10.1126/sciadv.aaw2880.
[35] Haertle, L.; Müller, T.; Lardenoije, R.; Maierhofer, A.; Dittrich, M.; Riemens, R.; Stora, S.; Roche, M.; Leber, M.; Riedel-Heller, S.; et al. Methylomic Profiling in Trisomy 21 Identifies Cognition- and Alzheimer's Disease-Related Dysregulation. *Clin. Epigenetics*, **2019**, *11*. https://doi.org/10.1186/s13148-019-0787-x.
[36] Jia, T.; Chu, C.; Liu, Y.; Dongen, J.; Papastergios, E.; Armstrong, N.; Bastin, M.; Carrillo-Roa, T.; Braber, A.; Harris, M.; et al. Epigenome-Wide Meta-Analysis of Blood DNA Methylation and Its Association with Subcortical Volumes: Findings from the ENIGMA Epigenetics Working Group. *Mol. Psychiatry*, **2019**, 1–12. https://doi.org/10.1038/s41380-019-0605-z.
[37] Dou, X.; Boyd-Kirkup, J.; McDermott, J.; Zhang, X.; Li, F.; Rong, B.; Zhang, R.; Miao, B.; Chen, P.; Cheng, H.; et al. The Strand-Biased Mitochondrial DNA Methylome and Its Regulation by DNMT3A. *Genome Res.*, **2019**, *29*. https://doi.org/10.1101/gr.234021.117.
[38] Johnson, L.; Torres, E.; Impey, S.; Stevens, J.; Raber, J. Apolipoprotein E4 and Insulin Resistance Interact to Impair Cognition and Alter the Epigenome and Metabolome. *Sci. Rep.*, **2017**, *7*, 43701. https://doi.org/10.1038/srep43701.
[39] Smith, A.; Smith, R.; Burrage, J.; Troakes, C.; Al-Sarraj, S.; Kalaria, R.; Sloan, C.; Robinson, A.; Mill, J.; Lunnon, K. A Cross-Brain-Regions Study of ANK1 DNA Methylation in 4 Different Neurodegenerative Diseases. *Neurobiol. Aging*, **2018**, *74*. https://doi.org/10.1016/j.neurobiolaging.2018.09.024.
[40] Pathak, G.; Silzer, T.; Sun, J.; Zhou, Z.; Daniel, A.; Johnson, L.; O'Bryant, S.; Phillips, N.; Barber, R. Genome-Wide Methylation of Mild Cognitive Impairment in Mexican Americans Highlights Genes Involved in Synaptic Transport, Alzheimer's Disease-

Precursor Phenotypes, and Metabolic Morbidities. *J. Alzheimer's Dis.*, **2019**, *72*, 1–17. https://doi.org/10.3233/JAD-190634.
[41] Fertan, E.; Rodrigues, G.; Wheeler, R.; Goguen, D.; Wong, A.; James, H.; Stadnyk, A.; Brown, R.; Weaver, I. Cognitive Decline, Cerebral-Spleen Tryptophan Metabolism, Oxidative Stress, Cytokine Production, and Regulation of the Txnip Gene in 3xTg-AD Mice. *Am. J. Pathol.*, **2019**, *189*. https://doi.org/10.1016/j.ajpath.2019.03.006.
[42] Mano, T.; Nagata, K.; Nonaka, T.; Tarutani, A.; Imamura, T.; Hashimoto, T.; Bannai, T.; Koshi-Mano, K.; Tsuchida, T.; Ohtomo, R.; et al. Neuron-Specific Methylome Analysis Reveals Epigenetic Regulation and Tau-Related Dysfunction of BRCA1 in Alzheimer's Disease. *Proc. Natl. Acad. Sci.*, **2017**, *114*, 201707151. https://doi.org/10.1073/pnas.1707151114.
[43] Semick, S.; Bharadwaj, R.; Collado-Torres, L.; Tao, R.; Shin, J.; Deep-Soboslay, A.; Weiss, J.; Weinberger, D.; Hyde, T.; Kleinman, J.; et al. *Integrated DNA Methylation and Gene Expression Profiling across Multiple Brain Regions Implicate Novel Genes in Alzheimer's Disease*; 2018. https://doi.org/10.1101/430603.
[44] Sanchez-Mut, J.; Heyn, H.; Silva, B.; Dixsaut, L.; Garcia-Esparcia, P.; Vidal, E.; Sayols, S.; Glauser, L.; Monteagudo, A.; Pérez-Tur, J.; et al. PM20D1 Is a Quantitative Trait Locus Associated with Alzheimer's Disease. *Nat. Med.*, **2018**, *24*. https://doi.org/10.1038/s41591-018-0013-y.
[45] CHATTERJEE, S.; Cassel, R.; Schneider Anthony, A.; Merienne, K.; Cosquer, B.; Tzeplaeff, L.; Sinha, S.; Kumar, M.; Chaturbedy, P.; Muthusamy, E.; et al. Reinstating Plasticity and Memory in a Tauopathy Mouse Model with an Acetyltransferase Activator. *EMBO Mol. Med.*, **2018**, *10*. https://doi.org/10.15252/emmm.201708587.
[46] Smith, A.; Smith, R.; Pishva, E.; Hannon, E.; Roubroeks, J.; Burrage, J.; Troakes, C.; Al-Sarraj, S.; Sloan, C.; Mill, J.; et al. Parallel Profiling of DNA Methylation and Hydroxymethylation Highlights Neuropathology-Associated Epigenetic Variation in Alzheimer's Disease. *Clin. Epigenetics*, **2019**, *11*. https://doi.org/10.1186/s13148-019-0636-y.
[47] Altuna Azkargorta, M.; Urdánoz-Casado, A.; Gordoa, J.; Zelaya, V.; Labarga, A.; Lepesant, J.; Roldán, M.; Blanco Luquin, I.; Perdones, Á.; Larumbe, R.; et al. DNA Methylation Signature of Human Hippocampus in Alzheimer's Disease Is Linked to Neurogenesis. *Clin. Epigenetics*, **2019**, *11*. https://doi.org/10.1186/s13148-019-0672-7.
[48] Dyer, M.; Phipps, A.; Mitew, S.; Taberlay, P.; Woodhouse, A. Age, but Not Amyloidosis, Induced Changes in Global Levels of Histone Modifications in Susceptible and Disease-Resistant Neurons in Alzheimer's Disease Model MiceData_Sheet_1.DocxImage_1.JPEGImage_2.JPEGImage_3.JPEGImage_4.JPEGImage_5.JPEGImage_6.JPEG. *Front. Aging Neurosci.*, **2019**, *11*. https://doi.org/10.3389/fnagi.2019.00068.
[49] Hernandez Hincapié, H.; Sandoval-Hernandez, A. Alzheimer's Disease DNA Methylome of Pyramidal Layers in Frontal Cortex: Laser-Assisted Microdissection Study. *Epigenomics*, **2018**, *10*. https://doi.org/10.2217/epi-2017-0160.
[50] Lee, M.; Lee, J.; Hyeon, S.; Cho, H.; Hwang, Y. J.; Shin, J.; Mckee, A.; Kowall, N.; Kim, J.; Stein, T.; et al. Epigenome Signatures Landscaped by Histone H3K9me3 Are Associated with the Synaptic Dysfunction in Alzheimer's Disease. *Aging Cell*, **2020**, *19*. https://doi.org/10.1111/acel.13153.
[51] Kaut, O.; Ramirez, A.; Pieper, H.; Schmitt, I.; Jessen, F.; Wüllner, U. DNA

Methylation of the TNF-α Promoter Region in Peripheral Blood Monocytes and the Cortex of Human Alzheimer's Disease Patients. *Dement. Geriatr. Cogn. Disord.*, **2014**, *38*, 10–15. https://doi.org/10.1159/000357126.
[52] Bernstein, A.; Lin, Y.; Street, C.; Lin, L.; Dai, Q.; Yu, L.; Bao, H.; Gearing, M.; Lah, J.; Nelson, P.; et al. 5-Hydroxymethylation-Associated Epigenetic Modifiers of Alzheimer's Disease Modulate Tau-Induced Neurotoxicity. *Hum. Mol. Genet.*, **2016**, *25*, ddw109. https://doi.org/10.1093/hmg/ddw109.
[53] Gao, Z.; Fu, H.; Zhao, L.; Sun, Z.; Yang, Y.; Zhu, H. Aberrant DNA Methylation Associated with Alzheimer's Disease in the Superior Temporal Gyrus. *Exp. Ther. Med.*, **2017**, *15*. https://doi.org/10.3892/etm.2017.5394.
[54] Vasanthakumar, A.; Davis, J.; Idler, K.; Waring, J.; Asque, E.; Riley-Gillis, B.; Grosskurth, S.; Srivastava, G.; Kim, S.; Nho, K.; et al. Harnessing Peripheral DNA Methylation Differences in the Alzheimer's Disease Neuroimaging Initiative (ADNI) to Reveal Novel Biomarkers of Disease. *Clin. Epigenetics*, **2020**, *12*. https://doi.org/10.1186/s13148-020-00864-y.
[55] Stoccoro, A.; Tannorella, P.; Migliore, L.; Coppedè, F. Polymorphisms of Genes Required for Methionine Synthesis and DNA Methylation Influence Mitochondrial DNA Methylation. *Epigenomics*, **2020**, *12*. https://doi.org/10.2217/epi-2020-0041.
[56] Fransquet, P.; Lacaze, P.; Saffery, R.; Phung, J.; Parker, E.; Shah, R.; Murray, A.; Woods, R.; Ryan, J. Blood DNA Methylation Signatures to Detect Dementia Prior to Overt Clinical Symptoms. *Alzheimer's Dement. Diagnosis, Assess. Dis. Monit.*, **2020**, *12*. https://doi.org/10.1002/dad2.12056.
